# When I Am Old: The Self-Face Recognition Advantage Disappears for Old Self-Faces

**DOI:** 10.3389/fpsyg.2019.01644

**Published:** 2019-07-29

**Authors:** Ronghua Zhang, Aibao Zhou

**Affiliations:** ^1^School of Psychology, Northwest Normal University, Lanzhou, China; ^2^Key Laboratory of Behavior and Mental Health of Gansu Province, Northwest Normal University, Lanzhou, China

**Keywords:** self-face, aging, self-reference effect, old-face recognition, IPA (implicit positive association)

## Abstract

The self-face is the unique representation of oneself, and it has a processing advantage over familiar faces and the faces of strangers. Generally, recognition of the self-face is significantly faster or more accurate in a variety of tasks compared with recognizing others’ faces. While previous studies that used the present self-face as stimuli have found a processing advantage, what happens when the self-face turns old? To investigate whether an old self-face can still produce the processing advantage, we conducted two experiments. Experiment 1 used a standard visual search paradigm, and Experiment 2 used the implicit association test. In both experiments, the old self-face was compared with the present self-face or an old friend-face. We found that when the self-face turns old, the processing advantage disappears. This research demonstrates a new way to investigate the future self.

The world’s older population continues to grow at an unprecedented rate. According to a report by the National Institutes of Health, in 2016, 8.5% of people worldwide (617 million) were aged 65 years and over, and this figure might reach 17% (1.6 billion) by 2050 (NIH-Funded Census Bureau, 2016). A large number of research studies have focused on how people recognize an old person. However, how people recognize the aging self is of interest in the current research study; that is, do people have a cognitive advantage relating to awareness of the aging self? The present study aimed to investigate whether the self-face, which is a unique representation of the self, still has a processing advantage when the self gets old.

The self-reference effect is a robust effect (Rogers et al., 1977) that indicates that the self-structure is unique, and it has a cognitive advantage in terms of a wide range of motivational, affective, and mnemonic consequences (Symons and Johnson, 1997). As a representation of the self, one’s face transmits information about oneself, which is rarely shared by others. Previous research has provided evidence that human adults respond faster to their own face than to the faces of others at a behavioral level (Keenan et al., 1999; Tong and Nakayama, 1999; Sui and Han, 2007) and that it activates unique brain regions at the neuropsychological level (Uddin et al., 2005; Kaplan et al., 2008). Compared with other-face stimuli, self-face perception may be correlated with neural activity in the right hemisphere (Keenan et al., 1999). Uddin et al. (2005) also found that a neural network involving the right hemisphere structures, which has mirroring properties and includes the inferior frontal gyrus and the inferior parietal lobule, is activated in self-face recognition.

To explain the response advantage in self-face recognition, Ma and Han (2009) proposed the implicit positive association theory. They believe that self-face perception activates positive attributes of the self-concept, which facilitates behavioral responses to the self-face and results in the self-advantage in face recognition. According to the theory, self-face recognition is strongly modulated by social threats. It has been found that participants respond more slowly to the self-face when a faculty advisor’s face (i.e., a high-threat context) is presented, compared with when a face of another faculty member (i.e., a low-threat context) and the self-face are presented (Ma and Han, 2009).

Aging can be regarded as a threat to oneself. Most research studies of age-related attitudes that use explicit measures have suggested that people’s perceptions of elderly adults are mixed; they associate both negative and positive traits with older people (Hummert et al., 1995). A large-scale international study of the stereotype content model found that participants from Belgium, Costa Rica, Hong Kong, Japan, Israel, and South Korea gave warmth scores for elderly groups of people that were significantly higher than the mean score for the overall warmth and gave competence scores that were significantly lower than the overall competence mean scores (Cuddy et al., 2005). In contrast to the evidence obtained using explicit measures, most of the implicit measures used in research studies of aging groups have found negative aging stereotypes (for a review, see Levy, 2003). Aging self-stereotypes explain the correlation between aging and a negative self-concept. Younger individuals tend to adopt their prejudice toward aging from their family or cultural environment, and they regard old-age groups as helpless, incapable of caring for themselves, and generally passive. These ageist perceptions may result in individuals morphing into these stereotypes themselves.

However, the question remains of whether an old self-face can engender a self-related information processing advantage. Since self-face was related to positive attribute, which shortens the process reaction time, while aging can be considered as a threaten for oneself. It is unknown whether people still connect positive attribute to themselves when aging, which itself contain negative attributes. It is a new perspective to see the self-face advantage processing. If participants process old self-face better than process old-other (i.e., friend) face, it maybe the evidence that self-face advantage is robust that can last till the aging lifetime; otherwise, the disappearance of old self-face may imply the negative attitude that oneself think about the aging self. Though numerous research focus on the aging people, few is about how people think of the aging self when they are young. Perceive or even interact with the aging self can dramatically influence the long-term choices that individuals make (Hershfield et al., 2011), thus, to understand the way we regard aging self may help explain and predict the behavior for aging, it is also important for the *well preparation* for aging.

We conducted two experiments to investigate old self-face recognition. Experiment 1 used a visual search paradigm (Tong and Nakayama, 1999) in which participants were required to respond quickly to target faces that included faces of the self, friends, and strangers at present and in old age. We expected that the old self-face would not induce a self-reference effect; hence, the reaction time (RT) of the old self-face would not be faster than that of the friends’ or strangers’ old faces. Experiment 2 adopted the implicit association test (IAT; Ma and Han, 2009) in which participants were required to connect positive or negative words with the present or old self-/friend-face. We predicted that the old self-face would have a more robust connection with the negative words than with the positive words.

## Experiment 1

### Method

#### Participants and Design

Forty right-handed participants [mean age: 23.33, standard deviation (SD) = 3.38, 18 males] were recruited in this study, all of whom were undergraduate and graduate students of Northwest Normal University. To create pairs, they were gender-matched with a friend they had known for more than 1 year (mean relationship duration = 20 months) and with whom they met more than five times per week (the pairs were made up of roommates; in addition to 20 one-to-one pairs, there were two triads. In each triad, there include three participants who knew each other: pair 1 includes students A and B; pair 2 includes students A and C; and pair 3 includes students B and C). The picture of stranger was randomly selected from all the participants’ pool who has not known by the participants. All participants were healthy, with normal or corrected-to-normal vision. All participants gave their written informed consent for the experiment. The Ethical Committee of School of Psychology, Northwest Normal University approved the study. The study used a 3 (person: self, friend, or stranger) × 2 (age: present or old) within-participants design.

#### Apparatus and Stimuli

All experiments were conducted on a Lenovo R4900d computer (Beijing, China) and presented on a 14-inch cathode ray tube monitor (120 × 150 pixels) with 256 gray levels. The stimuli were photos of faces that were individually tailored for each participant. Two different categories of stimuli were used: (1) a present face, which was a front-view image of each participant (the participants did not wear their glasses in all of the photos, and the photos were taken under controlled room lighting and with a white background) and (2) an old face, which was the present face transformed into an old face using FaceApp (2016 Wireless Lab OOO, 2016) on a smart cellphone. In order to minimize obvious visual cues for recognizing the face, the external features (hair and ears) were removed. All photos were stored with 256 gray levels and matched for size (120 × 150 pixels) within a fixed window size.

To confirm that the participants did regard the old face photo as being of themselves or their friends, they were required to rate the photos of the old self and the friends from 1 (*totally agree*) to 7 (*totally disagree*) and to state the age of the person in the photos using the following statements:

1. I think it is a photo of a real person (it does not look machine-made).
2. How old do you think the person in the photo is?

After the rating, all participants were told that there is a new technique for changing one’s present face into an old one. Then, we asked the participants to recheck the old photos and to confirm that the old photo was transformed from the present one.

The mean score for the statement about the realness of the old self-face was 2.55 (SD = 1.17), and it was 2.25 (SD = 1.08) for the old friend-face. The mean age that the participants gave for the old face was 63.70 years (SD = 9.96) for the self-face and 59.37 years (SD = 9.07) for the friend-face. The participants rated the old face as significantly older than their real present age, and the face transformed by FaceApp was accepted as real.

#### Procedure

**Figure 1:**
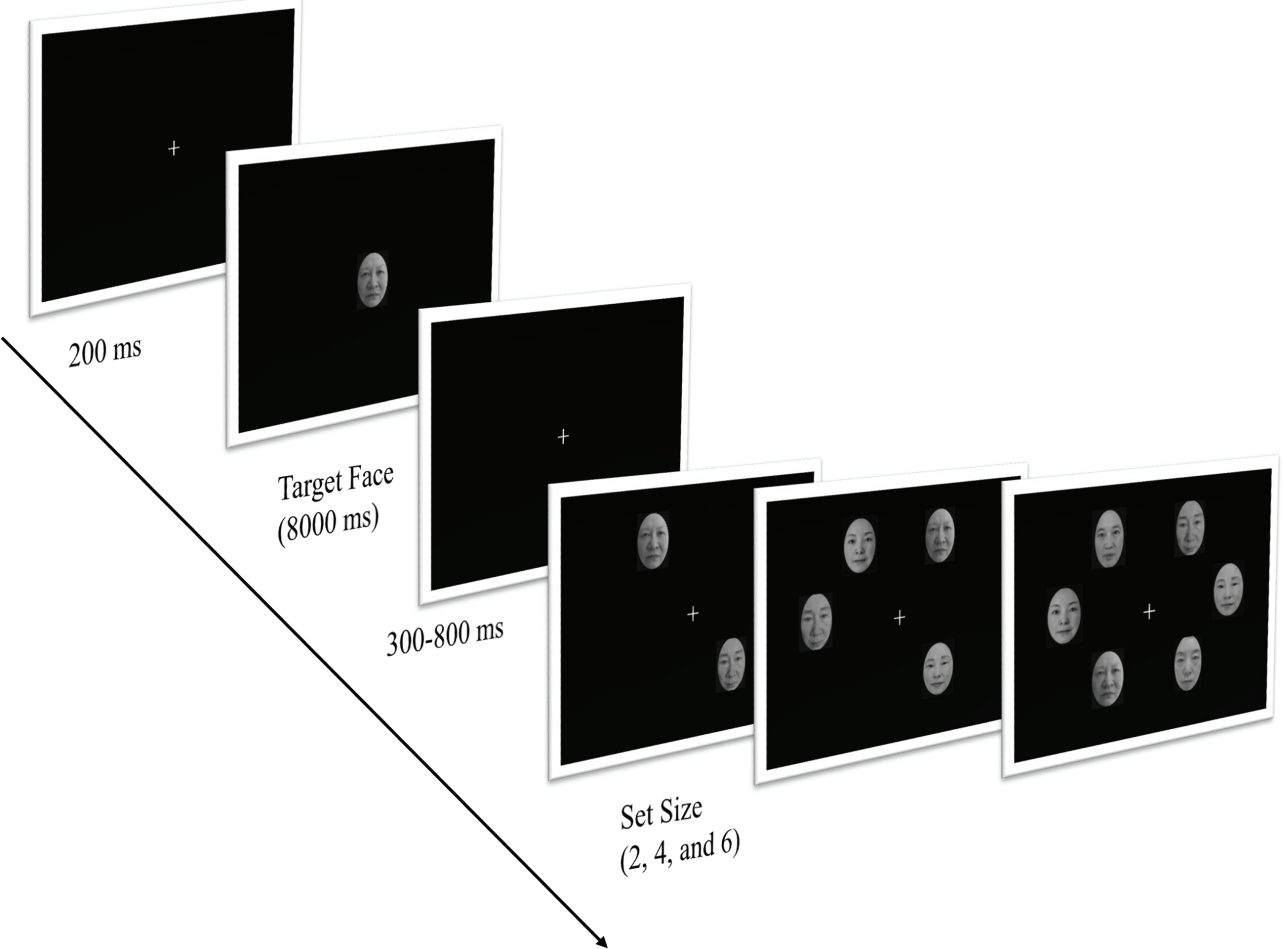
The procedure of Experiment 1.

A standard visual search paradigm (Tong and Nakayama, 1999) was used in Experiment 1. Participants were required to search for a target face in sets of two, four, or six faces. The target face was the present or old self-face, present or old friend’s face, and present or old strangers’ faces in six separate blocks. The order of the six conditions was counterbalanced using a random-block design. In each block, the target face was shown at the beginning once of each block, and it was followed by 108 test trials. On each trial, an array of two, four, or six faces was displayed after a fixation cross randomly appeared for 300–800 ms. In all, each block consisted of 36 trials (33%) of the two faces set size, 36 trials (33%) of the four faces set size, and 36 trials (33%) of the six faces set size. The distractor faces consisted of *n* (if there was no target face in the face set) or *n* – 1 faces (if the target face was in the face set; *n* = 2, 4, and 6 faces), which were randomly selected from the set of seven pictures (including one self-face, one friend-face, and five stranger faces; if the target was present face, then all the distractor faces were present faces; if the target was old face, all the distractor faces were old faces; the procedure of Experiment 1 was shown in Figure 1). In each block, the target was appeared on 50% of the trials. In order to control the left-hand advantage for self-face recognition, half of the participants responded “target present” by pressing “F” on the computer keyboard with their left hand and “target absent” by pressing “J” with their right hand; the other half of the participants responded “target present” by pressing “J” with their right hand and “target absent” by pressing “F” with their left hand.

#### Data Analysis

The reaction time (RT) and correct rate were analyzed. In order to detect potential speed-accuracy trade-offs, the correct rates were checked. The error rate for 40 participants was 9.2% (9.2% in 2 set, 8.6% in 4 set, and 9.9% in 6 set; SD = 3.84%); the participants’ correct rate and reaction time that was with more than 3 SD were removed. Finally, 36 participants’ data were analyzed. The mean overall error rate was 9.2% (SD = 3.66%), and there was no evidence of a speed-accuracy trade-off. A 3 (person: self, friend, or stranger) × 2 (age: present or old) within-subjects analysis of variance (ANOVA) was performed separately for the three set sizes (2, 4, and 6) for the correct responses.

### Results and Discussion

**Figure 2:**
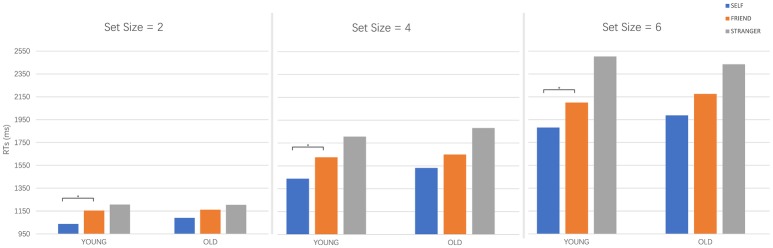
The mean reaction times of the present face and old face for the self, friends, and strangers in the two, four, and six faces set sizes. During the present condition, self-face was responded to significantly faster than the friend-face; while in the old condition, the reaction time for the self-face was not significantly different with the friend-face.

Figure 2 shows the mean RTs. Mauchly’s test indicated that the assumption of sphericity had been satisfied in the main effect of person and the interaction effect for the three set sizes separately (*χ*^2^_(2)s_ = 5.462, *p* = 0.065). A significant main effect of person was found for the three set sizes (set size 2: *F*(2, 70) = 5.687, *p* = 0.005, *η*^2^ = 0.140; set size 4: *F*(2, 70) = 17.032, *p* < 0.001, *η*^2^ = 0.327; set size 6: *F*(2, 70) = 18.803, *p* < 0.001, *η*^2^ = 0.349). *Post hoc* comparisons with Bonferroni correction indicated that the self-face responses were marginal significantly faster than the friend-face (*p*_s_ = 0.079, $\eta_{s}^{2}$ = 0.286) and stranger-face (*p*_s_ < 0.001, $\eta_{s}^{2}$ = 0.340) for all three set sizes, in which the friend-face responses were significantly faster than the stranger-face responses for the four and six faces set sizes (*p*_s_ = 0.013, $\eta_{s}^{2}$ = 0.543). The analyses showed no significant differences for age in set sizes (*F*_s_(1, 35) = 2.648, *p* = 0.113, *η*^2^ = 0.070).

The interaction between person and age was not significant for all three set sizes, while the further simple main effects comparisons revealed that for the target present condition, the self-face responses were faster than the stranger-face responses (*p*_s_ = 0.004, Cohen’s *d* = 0.521) and friend-face response (*p*_s_ = 0.034, Cohen’s *d* = 0.368) in all three set sizes; the friend-face responses were faster than the stranger-face responses in the four and six faces set sizes, (*p*_s_ = 0.034, Cohen’s *d* = 0.370). For the old condition, as in the present condition, the self-face was significantly faster than the stranger-face responses in all three set sizes (*p*_s_ = 0.026, Cohen’s *d* = 0.368); and friend-face responses were significantly faster than the stranger-face responses in the four and six faces set sizes (*p*_s_ = 0.033, Cohen’s *d* = 0.369); while the self-face response was not significantly different to the friend-face response in all three set sizes (*p*_s_ = 0.077, Cohen’s *d* = 0.277). In other words, the self-face processing advantage only appeared in the present condition; while for the old face condition, self-face showed no difference between friend-face.

Though the *p* of the interaction effect was not reach significant (below 0.05), the Cohen’s *d* showed that the simple main effect reached an acceptable medium size (Cohen, 1988), which indicates the difference reliable. Experiment 1 showed significantly faster responses to the present self-face than to the friend-face, and no significant difference between friend and self-face in the old condition, demonstrating that the self-reference effect disappears when the self becomes old. The self-reference effect is a robust cognitive bias, and it is evidence that the self-structure is unique, is processed elaborately, and has motivational implications that cause the faster RT or higher accuracy rate when there is recognition of self-reference information (Symons and Johnson, 1997). A great amount of research has shown this self-face processing advantage (Keenan et al., 1999; Tong and Nakayama, 1999; Sui and Han, 2007). The present experiment showed that the self-reference information processing has an advantage for just the present age and that when processing the old self-face, the advantage disappears.

As in previous research, our results showed that the present self-face RTs were significantly faster than the friend and stranger face RTs (Tong and Nakayama, 1999) in the two, four, and six faces set sizes, which indicate a salient self-reference advantage. Previous research has shown inconsistent results when comparing self-faces and familiar faces. Some studies found a self-advantage, indicating that self-face recognition is significantly faster than familiar-face recognition. They regarded the self-face as a specific stimulus that is represented more strongly and more robustly than the familiar-face and that it is processed in a unique way. Besides, familiarity is also a factor that affects the processing. Caharel et al. (2002) used P2 to explore the recognition of the different faces, and they found that a degree of familiarity effect occurred approximately 250 ms after stimulus onset, compatible with the idea that face representations are enriched with extensive visual experience. However, other studies have found that the self-face RT was not different to that of familiar faces, such as friends, siblings, or lovers (for a review, see Bortolon and Raffard, 2018). Bortolon et al. (2017) attribute these inconsistencies to the heterogeneity in the methodology and culture difference. The type of task, stimuli, or friendship between the familiar person and the self leads to the various results; and the participants from Western cultures tend to respond faster to their own face than to other people’s faces, while for participants from Eastern cultures, the effect was not significant. In the present research, the age might be one reason that attributes the inconsistency of self and friend-face recognition.

As hypothesized, the old self-face cannot induce the self-advantage. One possible explanation is that the old face has a less robust representation than the present face. In one meta-analysis, Rhodes and Anastasi (2012) found that there is an own-age bias (OAB) in face recognition, which confirms that people, including children, younger adults, and older adults, exhibit superior discriminability for the same-age face compared with other-age faces, showed a better memory for individuals of one’s owe age group compared with individuals of another age group. The contact and the recent experience of the perception of one’s own age ingroup members enhanced the OAB. Result of Experiment 1 showed significantly faster RTs for the familiar faces, including self and friend faces, than strangers’ faces in both present and old conditions, indicating that participants regard one’s own and friend’ face as familiar faces for both present an old condition. However, for the old condition, the self-face recognition advantage disappeared compared with friend-face. A previous study showed that exposure to one’s face results in more robust mental representations of the face, since it can facilitate the face processing by relying on structural codes that constitute the face recognition unit (White et al., 2016). Compared with the present self-face and friend-face that are familiar, the old self-face and old friend-face have never been seen in reality, though they can be recognized faster than old stranger face. Hence, the lack of exposure to the old face might lead no difference of representation of one’s own and friend faces, which will eliminate the advantage of self-related information.

Since there is no significant difference between the recognition of present and old faces, it has limitations to explain the disappearance of the old self-face recognition through exposure rate and experience. As the self is a unique representation (Keenan et al., 1999), the recognition of present one’s own face was significantly faster than the recognition of friend’s face, though they were both regarded as overlearned and familiar faces. One other alternative explanation for the self-face advantage is that the self-face or a loved familiar-face arouses stronger emotion than a stranger’s face (Guerra et al., 2012). The old self-face might induce negative emotion rather than positive emotion, since aging is always connected with a negative stereotype (Levy, 2003). Ma and Han (2009) proposed a theory to explain the self-face advantage that argues that the self-face activates an implicit positive association and results in a faster response. According to this theory, when the self-face turns old, it can activate a negative association and become a threat, and the self-face advantage will disappear. To further verify the disappearance of the positive bias to the self when processing an old self-face, we employed the IAT, as used by Ma and Han (2009), to compare the different attitudes toward the present self and old self.

## Experiment 2

### Method

According to Experiment 1, the old self-face did not show a recognition advantage as did the present self-face, one possible reason is that the age of the face disrupted the implicit association between the self-face and positive attributes. To further investigate the possible explanation of the disappearance of the self-face recognition advantage in old age, Experiment 2 adopted the IAT procedure to confirm the association between the old self-face and positive attributes. We hypothesized that the old self-face would not be associated with positive attributes as is the present self-face; hence, the RT for associating the old self-face with positive attributes would not be shorter than the RT for associating with negative attributes, while for the present face, as previous research has shown, associating the self-face with positive attributes would be faster than associating with negative attributes.

#### Participants and Design

Forty-one right-handed participants (mean age: 23.61, SD = 2.22 years, eight males) were recruited in this study. As in Experiment 1, all the participants were gender-matched into pairs with a friend they had known for more than 1 year (mean relationship duration = 20 months) and who they met more than five times per week (the pairs were made up of roommates; as there were three roommates in one case, the number of participants was uneven). All participants were healthy, with normal or corrected-to-normal vision. All participants gave their written informed consent for the experiment. The Ethical Committee of School of Psychology, Northwest Normal University approved the study.

#### Apparatus and Stimuli

The equipment and stimuli were the same as in Experiment 1.

#### Procedure

Experiment 2 consisted of two standard IAT procedures (Greenwald and Farnham, 2000). In the first procedure, which was similar to that of Ma and Han (2009), there were four kinds of stimuli, including *me* and *not me* items, which consisted of each participant’s present face (me) and a friend’s face (not me) and a positive item and negative item. The face image had a neutral expression, and it was taken before the experiment, as in Experiment 1. As in Ma and Han’s (2009) procedure, the stimuli consisted of 10 face images oriented to the left (from 0 to 45°) and to the right (from 0 to 45°). The positive and negative items both consisted of 10 words (Zhou et al., 2013). The second procedure was the same as the procedure of the first IAT procedure, except that we used the participants’ old face images as the me and not me items.

In the procedure, the participants had to categorize the items that appeared in the center of the screen. As in the previous paradigm, each IAT procedure consisted of seven blocks, five of which were practice blocks that consisted of 20 trials, and two of which were data collection blocks that consisted of 20 and 30 trials (Table 1). In each block, after the instruction, the stimuli appeared in the center of the screen followed by a fixation cross that lasted for 800–1,200 ms (*M* = 1,000 ms). Participants responded by pressing the “F” key with the left index finger or the “J” key with the right index finger. The assignment of different items to the left hand and right hand and the responses were counterbalanced across participants.

**Table 1:** The order of the procedure in the implicit association test.

Block and number of trials	Category 1	Category 2	Attribute
1: Practice, 20 trials	Me	Not me	
2: Practice, 20 trials	Positive	Negative	
3: Practice, 20 trials	Me + positive	Not me + negative	
**4: Data collection, 20 trials**	**Me + positive**	**Not me + negative**	**Congruent**
5: Practice, 20 trials	Negative	Positive	
6: Practice, 20 trials	Me + negative	Not me + Positive	
**7: Data collection, 30 trials**	**Me + negative**	**Not me + positive**	**Incongruent**

*Note: In Procedure 1, “me” represented the present self-face picture, and “not me” represented the present friend picture. In Procedure 2, “me” represented the old self-face picture, and “not me” represented the old self-face picture. The positive and negative categories each consisted of 10 adjectives. Based on a previous study, an individual always associates himself/herself with a positive attribute; thus, we used “me + positive” and “not me + negative” as the congruent condition, and “me + negative” and “not me + positive” as the incongruent condition*.

#### Data Analysis

The RTs of the correct responses and error rates were analyzed. The error rate for 41 participants was 1.6% (SD = 0.48); the participants’ data that had more than a 40% error rate and reaction time that was with more than 3 SD were removed. Finally, 36 participants’ data were analyzed, the mean overall error rate was 1.6% (SD = 0.47). A 2 (age: young or old) × 2 (person: self or friend) × 2 (association: congruent or incongruent) within-subjects ANOVA was performed. For the association condition, combinations of me + positive and of not me + negative were congruent, while the incongruent condition presented the combination of me + negative and of not me + positive.

### Results and Discussion

**Figure 3:**
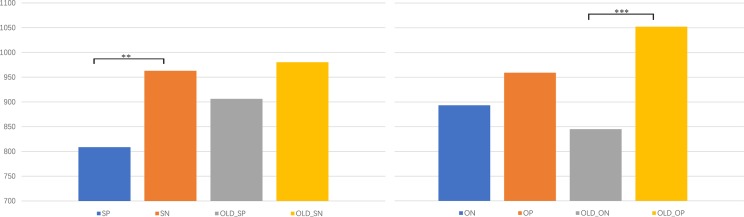
The mean reaction times of the self-face and friend-face associated with positive and negative words in the present and old face conditions. SP refers to self-positive; SN refers to self-negative; OP refers to other-positive; ON refers to other-negative; OLD refers to stimuli in old condition. The two kinds of faces showed different patterns. **p < 0.01; ***p < 0.001.

Table 2 shows the mean RTs. The ANOVAs of the RTs showed that there was a reliable significant interaction effect of age × person × association, *F*(1, 35) = 6.744, *p* = 0.014, *η*^2^ = 0.162; *post hoc* analysis confirmed that in self condition, participants responded faster to the self-positive than self-negative trials in the young condition, *F*(1, 35) = 11.728, *p* = 0.002, *η*^2^ = 0.251; whereas RTs did not show significant difference between positive and negative self-association in the old condition, *F*(1, 35) = 1.491, *p* = 0.23, *η*^2^ = 0.041; the results showed a different pattern for the friend condition, and there is no significant difference between positive and negative friend-association in the young condition, *F*(1, 35) = 2.600, *p* = 0.12, *η*^2^ = 0.069, whereas participants responded significantly faster to the friend-negative than friend-positive trials in the old condition, *F*(1, 35) = 26.228, *p* < 0.001, *η*^2^ = 0.428. The ANOVAs of the RTs showed a significant main effect of the association, *F*(1, 35) = 22.080, *p* < 0.001, *η*^2^ = 0.387; participants responded faster to the congruent association (*M* = 863.206, SD = 34.936) than to the incongruent association (*M* = 988.596, SD = 44.447; the results were shown in Figure 3).

**Table 2:** The mean (SD) of Experiment 2.

			*M* (RTs)	SD	*F*	*M* (ACC)	SD	*F*
Self	Young	Positive association	808.52	241.710	11.728**	99.17	2.803	1.103
		Negative association	962.81	308.945		98.33	3.695	
	Old	Positive association	906.21	315.811	1.491	98.89	3.187	0.264
		Negative association	980.08	323.766		98.70	3.490	
Friend	Young	Positive association	959.25	298.812	2.600	97.78	4.216	0.190
		Negative association	893.16	270.061		97.59	5.322	
	Old	Positive association	1052.13	355.048	26.228***	97.78	4.216	−0.723
		Negative association	844.93	221.786		98.33	3.333	

** *p < 0.01*.

*** p < 0.001;

As hypothesized, there was a different processing pattern for the old faces compared with the present faces. For the present face, when the self-face was associated with positive words, the RTs were significantly faster than when it was associated with negative words, indicating that people tend to associate positive information with themselves; whereas the friend face showed no significant difference when associated with positive and negative words. However, for the old self-face recognition, there was no significant difference between the association with positive and negative words; whereas participants responded significantly faster when associate negative than positive words with old friend-face. Consistent with Ma and Han (2009), the present experiment verified the implicit positive association with the self-face. The present self-face was easily associated with positive words since the participants connect the self with positive information. While the old self-face did not show this preference for positive words.

One explanation might be that aging eliminates the implicit positive association with the self-face. Aging is always associated with negative attributes both explicitly and implicitly, such as helplessness and not being able to take care of oneself. People are afraid of getting old as it is always related to a poor health condition, slower reactions, or poor memory (Hummert et al., 1995). Hence, when participants are required to connect the old self-face with positive words, the contradiction broken down the recognition advantage; on the other hand, the friend face recognition pattern showed that for the old face, it is much easier to associate negative words than positive words, which suggested that the old face was associated with negative more than positive attribute. One other explanation is that the old self is viewed from the third-person perspective. MacRae et al. (2015) used an imagination paradigm and found that when the future is distant enough, people view a story from the third-person perspective even when they are the main actor in the imaginary story. Since the old face of the participants and their friends was extremely old, it will take decades to reach that age. Hence, it might be that, for the old face, participants recognize it only from the third-person perspective. This perspective can lessen the positive bias (Zhou et al., 2013), and it may be an attribute that leads to the lack of difference between the self-face and friend-face in the old condition.

## General Discussion

The present study conducted two experiments and found that the old self-face does not engender a processing advantage compared with the friend-face. The disappearance of the self-reference advantage for the old face is confirmed to be both explicit and implicit.

Old-face pictures were used as the stimuli to represent the self and a friend. All participants did regard the old-face picture as their own-face or as a friend-face even though it does not exist in reality. This is a new way to look at the self-reference advantage. For decades, the self-reference effect has been found to be robust in face recognition, but, so far, no research has examined the old or future self-face. How people regard the aging self has not been confirmed yet. The present research used new stimuli (the transformed pictures) that presented the old self directly to the participants. Adopting the paradigms used in both explicit and implicit cognitive research, the present research investigated the future self in a novel way.

## Limitations

Though it is innovative to use the old face as the stimulus representing the aging self or a friend, this study also has some limitations. That is, in the present research, the age of the old face could not be controlled. According to the participants’ ratings of the age of the old face, and as the SD showed, the span between the oldest and youngest faces was not narrow. However, there was no doubt that all the old faces were significantly different to the present faces. Hence, the present research used the word “old” to present the identities of the long-term future self, friends, and strangers. In future research, the old face should be controlled more specifically; for instance, being shown as the same age or almost the same age.

Another limitation of the present research is that it failed to detect a gender difference regarding oneself getting old. Since the old self-faces were visually dramatically different with the young face, it might arouse more negative emotions in female participants than in male participants. However, in the present research, it was not an aim to examine the gender difference when verifying the old self-face processing.

## Conclusion

Overall, our findings suggest that the self-face advantage does not occur for the old face during both explicit and implicit cognitive processing. This finding was consistent with the own age bias (Rhodes and Anastasi, 2012) and might be explained by different factors, such as the lack of exposure to the old face, the old face disrupting the implicit positive association with the self, and the old self-face being viewed from the third-person perspective.

## Data Availability

The raw data supporting the conclusions of this manuscript will be made available by the authors, without undue reservation, to any qualified researcher.

## Author Contributions

RZ and AZ contributed the conception and design of the study. RZ organized the database, performed the statistical analysis, and wrote the first draft of the manuscript. All authors contributed to manuscript revision, read, and approved the submitted version.

### Conflict of Interest Statement

The authors declare that the research was conducted in the absence of any commercial or financial relationships that could be construed as a potential conflict of interest.
